# Technique of Stepwise Intracranial Decompression Combined with External Ventricular Drainage Catheters Improves the Prognosis of Acute Post-Traumatic Cerebral Hemispheric Brain Swelling Patients

**DOI:** 10.3389/fnhum.2015.00535

**Published:** 2015-09-29

**Authors:** Lei Shi, Guan Sun, Chunfa Qian, Tianhong Pan, Xiaoliang Li, Shuguang Zhang, Zhimin Wang

**Affiliations:** ^1^Department of Neurosurgery, The First People’s Hospital of Kunshan Affiliated with Jiangsu University, Suzhou, China; ^2^Department of Neurosurgery, Fourth Affiliated Yancheng Hospital of Nantong University, Yancheng, China; ^3^Department of Neurosurgery, Nanjing Medical University Affiliated Nanjing Brain Hospital, Nanjing, China; ^4^Department of Neurosurgery, Suzhou Kowloon Hospital Affiliated with Shanghai Jiao Tong University School of Medicine, Suzhou, China

**Keywords:** stepwise intracranial decompression, external ventricular drainage, hemispheric brain swelling, GOS Score, severe traumatic brain injury

## Abstract

**Background:**

Acute post-traumatic cerebral hemispheric brain swelling (ACHS) is a serious disorder that occurs after traumatic brain injury, and it often requires immediate treatment. The aim of our clinical study was to assess the effects of stepwise intracranial decompression combined with external ventricular drainage (EVD) catheters on the prognosis of ACHS patients.

**Methods:**

A retrospective study was performed on 172 cases of severe craniocerebral trauma patients with ACHS. The patients were divided into two groups: unilateral stepwise standard large trauma craniectomy (S-SLTC) combined with EVD catheter implants (*n* = 86) and unilateral routine frontal temporal parietal SLTC (control group, *n* = 86).

**Result:**

No significant differences in age, sex, or pre-operative Glasgow Coma Scale score were observed between groups (*P* < 0.05). There were no significant differences in the ipsilateral subdural effusion incidence rates between the S-SLTC + EVD treatment group and the routine SLTC group. However, the incidence rates of intraoperative acute encephalocele and contralateral epidural and subdural hematoma in the S-SLTC + EVD group were significantly lower than those in the SLTC group (17.4 and 3.5 vs. 37.2 and 23.3%, respectively). The mean intracranial pressure (ICP) values of patients in the S-SLTC + EVD group were also lower than those in the SLTC group at days 1 through 7 (*P * < 0.05). A positive neurological outcome [Glasgow Outcome Scale (GOS) score 4–5, 50.0%] and decreased mortality (15.1%) was observed in the S-SLTC + EVD group compared to the neurological outcome (GOS score 4–5, 33.8%; 36.0%) in the SLTC group (*P * < 0.05).

**Conclusion:**

Our data suggest that S-SLTC + EVD is more effective for controlling ICP, improving neurological outcome, and decreasing mortality rate compared with routine SLTC.

## Introduction

Acute craniocerebral trauma is a severe clinical condition that commonly occurs in trauma patients. Brain edema and swelling often occur during traumatic brain injuries. Severe traumatic brain injury (STBI), defined as a head trauma that is associated with a Glasgow Coma Scale (GCS) score of 3–8, leads to increased mortality and morbidity and poses a major problem in critical care medicine (Teasdale and Jennett, 1974). STBI leads to extensive brain tissue contusion and secondary brain injury, which is characterized by brain tissue volume expansion and severe cerebral edema that ultimately results in intractable intracranial hypertension.

Post-traumatic acute brain swelling (PABS) is a severe complication that can occur after STBI. PABS is usually divided into two types based on the results of the CT image: acute generalized brain swelling (AGBS) and acute post-traumatic cerebral hemispheric brain swelling (ACHS). ACHS occurs in STBI patients with unilateral epidural hematoma and ipsilateral severe brain contusion and laceration. A high mortality is observed regardless of operation. Characteristics of ACHS include hemispheric swelling, a shifted midline, and ambient cistern compression or disappearance. ACHS is a serious problem after craniocerebral trauma. After the injury, the GCS score in ACHS patients sharply decreases to <8 points within a short time. In addition, the patients are afflicted by severe brain swelling and malignant intracranial hypertension. Factors involved in ACHS formation include cerebral microcirculation disturbance, cerebral vascular dilatation, cerebral ischemia and hypoxia, cerebral metabolic disorders, and accumulation of oxygen free radicals and acidic metabolites, which stimulate brain cells ischemia and swelling and worsen the damage of brain cells.

The rapid expansion of the cerebral vasculature after ACHS causes a rapid increase in brain volume, which is susceptible to intracranial hypertension and cerebral hernia formation and is difficult to treat. Without treatment, the subsequent increase in intracranial pressure (ICP) in addition to extensive swelling and aggravated cerebral contusion lead to the deterioration of patients with STBI. The best medical treatment combines sedation, dehydration, osmotherapy, blood pressure control, hyperventilation, and therapeutic hypothermia management. However, routine non-surgical methods are often used to control intracranial hypertension. Decompressive craniectomy (DC) is the primary method used to treat ICP. DC can prevent the increase in cranial volume and reduce ICP, thereby preventing the occurrence of cerebral hernia and restoring the cerebral blood flow perfusion. However, Chibbaro and Tacconi (2007) reported that after DC, 45% of STBI patients still had a Glasgow Outcome Scale (GOS) score of 3 or lower in a 14-month follow-up, suggesting that a considerable fraction of the patients still had a poor prognosis after routine DC. Acute intraoperative encephalocele is considered to be the leading cause for poor prognosis. A method to reduce and control intraoperative acute encephalocele can enhance the beneficial effects of an ACHS operation.

To reduce and control intraoperative acute encephalocele during an ACHS operational procedure, we implemented a novel technology that combines stepwise standard large trauma craniectomy (S-SLTC) with external ventricular drainage (EVD) catheter implants. The effects and the prognosis of STBI patients were observed and analyzed after the procedure.

## Materials and Methods

### General information

A total of 236 patients with ACHS injury admitted from March 2009 to March 2015 at our hospital were selected for this study. The exclusion criteria included (1) primary brain stem injury; (2) complications in vital organs, such as the heart, liver, lungs, and kidneys; (3) complications involving hemorrhagic shock, severe coagulation abnormalities, and multiple organ failure; (4) histories of brain tumors and cerebral infarction; (5) younger than 16 years old; (6) intracranial infection after the operation. One hundred seventy-two ACHS in-patient adults (S-SLTC + EVD treatment, 86 cases; SLTC treatment, 86 cases) were admitted into this study. All of the ACHS patients exhibited a midline shift of more than 5 mm. There were 129 males and 43 females with ages between 27 and 63 and 39.7 ± 12.3 years, respectively. Among the patients, the injuries were caused by traffic accidents (76 cases), falling (63 cases), combat (30 cases), and tumbling (3 cases). The time from injury to the start of operation was approximately 6 h. The GCS of each patient was eight or less at the time of admission; however, patients with a GCS score of 3 were not included in this study. A swollen hemisphere occurred at the right side for 117 cases and at the left side for 55 cases according to CT scans. Between the two groups, there were not any significant differences in sex, age, and pre-operative GCS. The study was approved by the ethics committee of the hospital, and informed consent forms were signed by the family members of the patients.

### Management procedures

Routine medical management, such as dehydration with 250 ml of 20% mannitol, was administered in the emergency room for herniation. Meanwhile, routine pharmacological or physical measures were used to maintain patient vital signs. Patients had subsequently undergone an immediate emergency CT examination. One to two hours after CT diagnosis, patients underwent craniectomy. Indications of emergency surgery included the following: (1) GCS < 8; (2) unilateral or bilateral mydriasis with positive pathologic reflex; (3) the third ventricle and basal cistern narrow midline shift >5 mm and unconsciousness.

### Surgery and treatment methods

Standard large trauma craniectomy surgery was undertaken as follows. Briefly, the bone window was approximately 14 by 10 cm with duraplasty using an expanded dura substitute when necessary. The anterior reached the frontal pole, and the posterior line was approximately 3–4 cm posterior to the external acoustic meatus. The superior line was 2 cm of the lateral edge of the central line, and the inferior line was extended 1 cm anterior to antilobium (Qiu et al., 2009). The bone window should reach middle cranial fossa to mitigate pressure on the center of the brain. Dura mater was exposed after a “Y” shape incision was made after removal of a bone flap. After removal of the hematoma or the contusion in the brain tissue, an artificial meningeal tissue was used as a dural tension-reduced suture. If necessary, a portion of the brain lobes was resected for the purpose of decompression. Finally, postoperative drainage tubes and intracranial monitoring pipes were placed.

**Figure 1:**
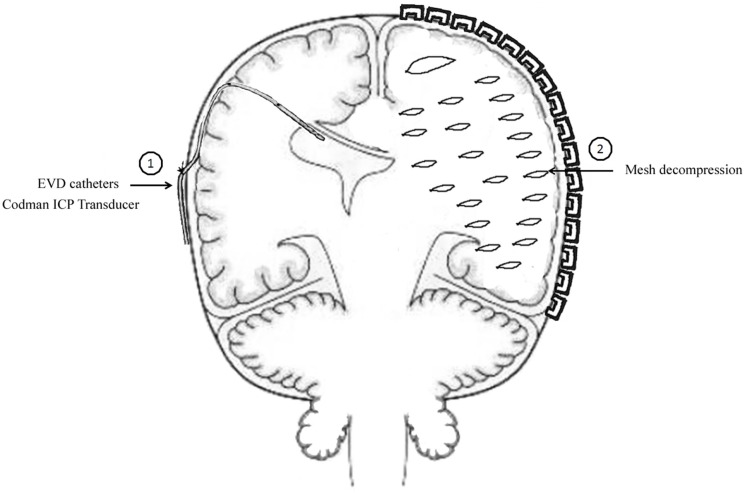
**Mode chart method for unilateral S-SLTC combined with contralateral external ventricular drainage (EVD) catheters implants**.

The method of unilateral S-SLTC combined with EVD catheter implants was proceed as follows. The contralateral frontal horn of the lateral ventricle was punctured, and the cerebrospinal fluid was drained before the S-SLTC operation. A frontal burr hole was placed 1 cm anterior to the coronal suture in the midpupillary line. Next, while using the Codman ICP Monitor system, which monitors ICP and drains cerebrospinal fluid, the catheter was inserted into the ventricle 4.0–5.5 cm from the cortex. After fixing the catheter, the S-SLTC operation was undertaken. By contrast, 1–2 incisions measuring 0.5–1 cm in length were made in the S-SLTC operation. After opening the dura, a suction tip was positioned at the epidural incision; if there was blood in the cerebrospinal fluid outflow or a hematoma, a small piece of cotton padding would have been placed at the incision and held against the suction tip to slowly reduce ICP and until no liquid drained out. The suction tip was subsequently inserted into the opening of the dural incision to remove the contusion and hematoma tissues. When the ICP dropped to 20–25 mmHg or less, the dura was opened, and the brain contusion and hematoma were further cleared. After complete hemostasis, compression hemostasis was performed with cotton padding. If the brain tissue pressure continued to be more than 25 mmHg during the stepwise decompression process, which led to high dural tension, a mesh-style cutting was performed on the dura, which was accompanied by intravenous drips of mannitol and furosemide and hyperventilation. The brain contusion and hematoma could later be removed through the mesh-style openings of the dura (Figure 1). When the ICP was below 25 mmHg during the cleaning, the dura was fully opened to clean out the brain contusion and hematoma. However, when the ICP was increasing over 25 mmHg, and when the brain tissue expelled out of the mesh-style dural opening after performing the above-mentioned stepwise decompression method, the brain contusion tissue and subdural hematoma were removed from the dural opening as much as possible according to the patient’s intraoperative vital signs. It should be noted that the dural opening was not fully opened.

### Postoperative therapy

**Figure 2:**
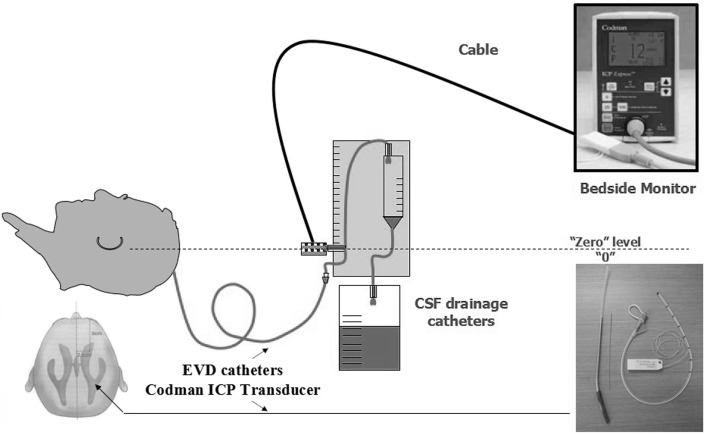
**Mode chart method for the Codman ICP monitor system**.

Intracranial pressure was continuously recorded using the Codman ICP monitor system in all patients for 7 days (Figure 2). The EVD catheter was used to control ICP, and the catheter was removed 7 days later. Postoperative drainage tubes were removed 48–72 h after the operation. Rational postoperative therapy included conventional medical steps (elevation of the upper part of the body by 15°–30° and maintenance of the respiratory tract patency), slight hyperventilation, sedation, if necessary, moderate hypothermia, controlling the blood glucose level, and balancing water and electrolyte levels. If ICP exceeded 20 mmHg, dehydration was administered. Early tracheotomy was also a necessary procedure.

### Observation items

The following parameters of the patients were recorded after craniectomy: (1) ipsilateral subdural effusion incidence rates; (2) incidence rates of intraoperative acute encephalocele; (3) incidence rates of contralateral delayed epidural and subdural hematoma; (4) postoperative mean ICP values of patients; (5) GOS scores. The following was used to determine GOS scores, which were evaluated at the 6-month follow-up after injury: (1) death; (2) persistent vegetative state; (3) severe disability; (4) moderate disability; (5) mild or no disability. In this retrospective study, the observed infection rates were 0.

### Statistical methods

SPSS 16.0 software was used in the analysis. For data analysis, mean ± SD was used. Intergroup comparisons were performed using the independent *t*-test. The chi-square test was used to analyze the numerical data. *P* < 0.05 was considered to be statistically significant.

## Results

### No significant differences were found in routine monitoring indexes

All patients with a GCS score of lower than 8 underwent tracheotomy after surgery. There were not any significant differences in routine monitoring indexes, including temperature, heart rate, respiration rate, and blood pressure 7 days after craniectomy between the S-SLTC + EVD group and the routine SLTC group (Table 1).

**Table 1:** **Clinical features of patients undergoing S-SLTC + EVD or SLTC treatment for ACHS**.

Observation index	S-SLTC + EVD	SLTC	*P*
*N*	86	86	
Age (years)	40.16 ± 13.2	39.22 ± 11.5	0.623
Sex			
Male	64	65	
Female	22	21	0.860
GCS			
>3, ≤5	40	37	
>5, ≤8	46	49	0.646
Pre-operative time (h)	6.3 ± 0.7	5.7 ± 0.3	0.536
Temperature (°C)	36.7 ± 0.3	36.6 ± 0.3	0.831
Heart rate	69.6 ± 14.1	71.0 ± 18.4	0.545
Respiration rate	18.3 ± 3.2	17.4 ± 3.4	0.078
Blood pressure (systolic pressure)	177.8 ± 13.4	174.6 ± 13.5	0.145

### S-SLTC + EVD treatment significantly reduced the incidence of intraoperative acute encephalocele and contralateral epidural and subdural hematoma in the early postoperative period

In routine DC, after the removal of intracranial hematoma, the contralateral dura is easily detached from the skull internal lamina or the bridging veins can be torn off, causing the formation of a contralateral epidural hematoma. Also, if there was a small amount of subdural hematoma on the contralateral brain, the contralateral subdural hematoma would increase rapidly when the surgical side of the dura was opened. Furthermore, removal of the hematoma resulted in a rapid decrease in ICP on the surgical side. Thus, the sharp decline of ICP was a major cause of intraoperative encephalocele. On the contrary, acute dilatation of cerebral vasculature was also considered to be a major cause of intraoperative acute encephalocele.

The greatest advantage of S-SLTC treatment is the gradual release of brain tissue pressure. Prior to the operation, EVD treatment could effectively decrease intraoperative high ICP state by releasing part of the cerebrospinal fluid to reduce the occurrence of contralateral hematoma and encephalocele. In this study, we determined that the incidence rate of intraoperative acute encephalocele was 17.4% in the S-SLTC + EVD treatment group compared with 37.2% in the routine SLTC group (*P* < 0.01). The incidence rate of contralateral epidural and subdural hematoma was 3.5% in the S-SLTC + EVD treatment group compared with 23.3% in the routine SLTC group (*P * < 0.01). However, no significant differences in ipsilateral subdural effusion incidence rates were found between the S-SLTC + EVD group and the routine SLTC group. These results demonstrated that SLTC + EVD treatment significantly reduced the incidence of intraoperative acute encephalocele and contralateral epidural and subdural hematoma compared with the routine SLTC treatment.

### S-SLTC + EVD treatment was conducive to early control of intracranial pressure

Postoperative ICP control is extremely important for improving the prognosis of traumatic brain injury (TBI) patients. Conventional dehydrate drugs include mannitol, albumin, and diuretic. The advantage of EVD is that it allows the simultaneous monitoring of ICP while draining the cerebrospinal fluid. The EVD of cerebrospinal fluid can be a good regulator of ICP because it reduces the use of mannitol and other dehydrate drugs. A large dose of mannitol can cause many side effects, such as mannitol-induced renal impairment and frank cardiac decompensation (Chen et al., 2007).

The mean ICP values of hemispheric brain swelling patients in the routine SLTC group could be controlled at 15.63 ± 3.21, 17.32 ± 1.27, 16.63 ± 2.17, 15.01 ± 2.07, 13.01 ± 1.70, 11.31 ± 1.32, and 9.63 ± 2.03 mmHg from 1 to 7 days, respectively. Our data showed that S-SLTC + EVD treatment could control ICP better than the routine SLTC treatment. The mean ICP values in the S-SLTC + EVD group could be controlled at 10.31 ± 2.13, 13.15 ± 1.26, 11.32 ± 1.07, 10.71 ± 1.32, 8.32 ± 1.67, 7.63 ± 1.63, and 7.36 ± 1.53 mmHg, respectively. The ICP differences between the two groups were statistically significant (*P * < 0.05). Also, we observed that the EVD device with ICP monitoring can rapidly control an ICP of <20 mmHg by drainage of cerebrospinal fluid.

### S-SLTC + EVD treatment improved the neurological outcomes of hemispheric brain swelling patients

With a mean follow-up of 6 months, 31 (36.0%) patients died in the postoperative period, 17 (19.8%) patients were in a persistent vegetative state with a GOS of 2, 9 (10.5%) patients were severely disabled with a GOS of 3, 20 (23.3%) patients were moderately disabled with a GOS of 4, and 9 (10.5%) patients had a GOS of 5 after rehabilitation in the routine SLTC group. Among the S-SLTC + EVD group, 13 (15.1%) had a GOS of 1, 15 (17.4%) had a GOS of 2, 15 (17.4%) had a GOS of 3, 26 (30.2%) had a GOS of 4, and 17 (19.8%) had a GOS of 5. A positive neurological recovery (GOS score 4–5) between the S-SLTC + EVD group and the routine SLTC group was significant (50.0 vs. 33.8%) (*P * < 0.05). Additionally, a lower mortality was observed in the S-SLTC + EVD group (15.1%) compared with that in the routine SLTC group (36.0%) (*P * < 0.01).

## Discussion

Acute hemispheric brain swelling is perhaps the most intriguing and least understood anatomical abnormality that occurs after severe TBI. This swelling is often characterized by uncontrollable intracranial hypertension, which leads to a high mortality rate. DC, a surgical procedure that involves the removal of a part of the skull to accommodate brain swelling, is an important method for the treatment of intracranial hypertension after craniocerebral trauma. A matched-pair analysis of long-term clinical outcomes in STBI patients with and without the DC technique in the Girotto et al. (2014) study demonstrated that DC could significantly reduce the mortality of STBI patients. These researchers determined that early DC within the first 24 h after the injury could reduce the mortality of STBI patients to 18% compared to the mortality of 35% in the matched control group without DC. DC is an effective method that removes the hematoma and contusive brain tissue that effectively reduces ICP and provides additional space for the swollen brain, thereby relieving the formation of cerebral hernia in patients.

Lü et al. (2003) reported that standard large trauma craniectomy (SLTC) could significantly reduce the mortality of STBI patients. SLTC creates a wider area of bone window compared with routine DC, including the frontal lobe, temporal lobe, parietal lobe, and anterior and middle cranial fossa. SLTC can greatly expose the brain contusion and laceration of the fontal and temporal pole, which is beneficial for removing the hematoma and necrotic brain tissue. Because of the lower edge of the bone window, this method can use a brain spatula to lift the temporal lobe to expose the cerebellar screen hole and can open the skull base brain pool to release the cerebrospinal fluid even further, alleviating a cerebral hernia. Lü et al. (2003) reported 230 patients with severe TBI and showed that a 41.7% mortality was found in the SLTC group compared with a 57.4% mortality in routine DC, demonstrating that SLTC can be more effective than routine DC to reduce the mortality of STBI patients. In this study, we showed that 26.7% of ACHS patients died after a SLTC operation in a mean follow-up of 6 months. The lower mortality rate after a SLTC operation may be related to the progress of the Neurosurgical Intensive Care Unit in recent years and the development of novel surgical instruments.

The aim of our study is to determine how the mortality rate can be reduced and to improve the prognosis of ACHS patients. Thus, we modified the operation of SLTC by including stepwise intracranial decompression methods. Because the formation of acute encephalocele and delayed hematoma often occurs during the rapid decompression process, we adopted a S-SLTC method. During the process of SLTC, opening the meninges often causes the brain tissue to expand rapidly out of the bone window, aggravating brain swelling and causing a midline reverse shift. A midline reverse shift can lead to a contralateral dural dissection from the skull and rupture the bridging vein, which causes the formation of acute encephalocele and delayed hematoma. Jiang et al. reported that they used a gradual and controlled decompression technique to effectively prevent and control massive intraoperative brain swelling. Their method focused on creating numerous small dural openings, making “U-shaped, discontinuous, small dural incisions” around the circumference of the craniectomy and making a dural relaxation suture (Jiang et al., 2014). The advantage of our technology is that an EVD is conducted and the ICP is monitored before craniectomy. Pre-operative EVD technology can reduce intracranial hypertension and relieve the process of intracranial cerebral hernia by pre-operative release of part of the cerebrospinal fluid. Whether the dural tissue can be opened after S-SLTC and when the dural tissue can be opened can be determined by ICP monitoring. Jiang et al. (2014) reported that within 6 months, the mortality rate was 37.5% in STBI patients after using a gradual and controlled decompression technique. Based on this study, the mortality rate was reduced to 15.1% within 6 months after S-SLTC + EVD operation. Thus, our combined technique of S-SLTC and EVD can improve the outcome.

In summary, the results of this study indicate that the combined technique of S-SLTC and EVD can prevent severe brain swelling and reduce complications, ultimately yielding a better outcome for ACHS patients.

## Conflict of Interest Statement

All authors have declared the sources of research funding for this manuscript and have no financial or other contractual agreements that might cause (or be perceived as causes of) conflicts of interest.

## Abbreviations

ACHS, acute post-traumatic cerebral hemispheric brain swelling; AGBS, acute generalized brain swelling; DC, decompressive craniectomy; EVD, external ventricular drainage; GCS, Glasgow Coma Scale; GOS, Glasgow Outcome Scale; ICP, intracranial pressure; PABS, post-traumatic acute brain swelling; SLTC, standard large trauma craniectomy; S-SLTC, stepwise standard large trauma craniectomy; TBI, traumatic brain injury.

## References

[B1] Chen C. F. Liu X. F. Meng X. Z. Jia H. Y. (2007). Comparative study of mannitol-induced acute kidney impairments in patients of different ages suffering from subarachnoid hemorrhage. Zhongguo Wei Zhong Bing Ji Jiu Yi Xue 19, 727–730. 18093429

[B2] Chibbaro S. Tacconi L. (2007). Role of decompressive craniectomy in the management of severe head injury with refractory cerebral edema and intractable intracranial pressure. Our experience with 48 cases. Surg. Neurol. 68, 632–638. 10.1016/j.surneu.2006.12.046 17765952

[B3] Girotto D. Ledić D. Daji V. Vujković Z. Mihelcić N. (2014). Neurosurgical procedure for treatment of traumatic subdural hematoma with severe brain injury: a single center matched-pair analysis. Coll. Antropol. 38, 1255–1258. 25842771

[B4] Jiang Y. Z. Lan Q. Wang Q. H. Song D. L. Lu H. Wu W. J. (2014). Gradual and controlled decompression for brain swelling due to severe head injury. Cell Biochem. Biophys. 69, 461–466. 10.1007/s12013-014-9818-6 24442991

[B5] Lü L. Q. Jiang J. Y. Yu M. K. Hou L. J. Chen Z. G. Zhang G. J. (2003). Standard large trauma craniectomy for severe traumatic brain injury. Chin. J. Traumatol. 6, 302–304. 14514369

[B6] Qiu W. Guo C. Shen H. Chen K. Wen L. Huang H. (2009). Effects of unilateral decompressive craniectomy on patients with unilateral acute post-traumatic brain swelling after severe traumatic brain injury. Crit. Care 13, R185. 10.1186/cc8178 19930556 PMC2811943

[B7] Teasdale G. Jennett B. (1974). Assessment of coma and impaired consciousness: a practical scale. Lancet 2, 81–84. 10.1016/S0140-6736(74)91639-0 4136544

